# *Dirofilaria immitis* and *Dirofilaria repens*: Investigating the Prevalence of Zoonotic Parasites in Dogs and Humans in a Hyperenzootic Area

**DOI:** 10.3390/ani14172529

**Published:** 2024-08-30

**Authors:** Dimitris Dimzas, Georgios Aindelis, Andronki Tamvakis, Sapfo Chatzoudi, Katerina Chlichlia, Maria Panopoulou, Anastasia Diakou

**Affiliations:** 1 Laboratory of Parasitology and Parasitic Diseases, School of Veterinary Medicine, Aristotle University of Thessaloniki, 54124 Thessaloniki, Greece; dimzas@vet.auth.gr; 2 Laboratory of Molecular Immunology, Department of Molecular Biology and Genetics, Democritus University of Thrace, 68100 Alexandroupolis, Greece; g.aindelis@gmail.com (G.A.); achlichl@mbg.duth.gr (K.C.); 3 Laboratory of Ecology and System Dynamics, Department of Marine Sciences, University of the Aegean, 81100 Mytilene, Greece; atamvaki@mar.aegean.gr; 4 Independent Researcher, Andreou Dimitriou 16, 67100 Xanthi, Greece; sapfochatzoudi@gmail.com; 5 Laboratory of Microbiology, School of Medicine, Democritus University of Thrace, 68100 Alexandroupolis, Greece; mpanopou@med.duth.gr; 6 Diagnostics and Laboratory Research Task Force, Balkan Association for Vector-Borne Diseases, 21000 Novi Sad, Serbia

**Keywords:** comparative epidemiology, dog, Greece, heartworm, human dirofilariosis, human, One Health, prevalence, pulmonary dirofilariosis, subcutaneous dirofilariosis, zoonosis

## Abstract

**Simple Summary:**

*Dirofilaria immitis* and *Dirofilaria repens* are mosquito-borne zoonotic parasites that primarily infect dogs. In recent years, the distribution of these parasites has expanded into previously non-enzootic areas. This study aimed to investigate the infection rates in dogs and humans within a hyperenzootic region of Europe, and to estimate the proportional relationship between infection prevalence in dogs and humans. Blood samples from 604 dogs and serum samples from 625 humans residing in Thrace, in northeastern Greece, were collected. The dog samples were examined for *Dirofilaria* spp. microfilariae using Kott’s test and for *D. immitis* antigen using a commercial serological test. The human sera were analyzed by Western blot for both parasites. Overall, 177 (29.3%) dogs were found to be infected, with 173 (28.6%) positive for *D. immitis* and 7 (1.2%) for *D. repens*, including 6 (1%) dogs with mixed infections. Specific IgG was detected in 42 (6.7%) human samples, with 24 (3.8%) positive for *D. immitis* and 18 (2.9%) for *D. repens*. The proportion of infection in humans was 23.4% of the corresponding canine infections in the study area, indicating a high risk of human infection in this hyperenzootic region.

**Abstract:**

The mosquito-borne zoonotic nematode parasites *Dirofilaria immitis* and *Dirofilaria repens* primarily affect dogs. In recent years, their distribution has expanded due to various factors influencing vector-borne pathogens. This study aimed to investigate the comparative prevalence of infection in dogs and humans within a hyperenzootic region of Europe, and to estimate the proportional relationship between infection prevalence in dogs and humans, within the concept of “One Health”. To this end, 604 blood samples from dogs and 625 serum samples from humans living in the Thrace region of northeastern Greece were collected. The dog samples were examined for *Dirofilaria* spp. microfilariae using Kott’s test and for *D. immitis* antigen using a commercial serological test. The human sera were analyzed for both parasites by Western blot. The overall prevalence of infection in dogs was 177 (29.3%), with 173 (28.6%) testing positive for *D. immitis* and 7 (1.2%) for *D. repens*, including 6 (1%) cases of mixed infection. Specific IgG antibodies were detected in 42 (6.7%) human samples, with 24 (3.8%) positive for *D. immitis* and 18 (2.9%) for *D. repens*. The infection proportion in humans was 23.4% of the corresponding canine infections, indicating a high risk of human infection in this hyperenzootic region.

## 1. Introduction

*Dirofilaria immitis* and *Dirofilaria repens* (Rhabditida: Onchocercidae) are nematode parasites transmitted by the bites of infected culicid mosquitoes. While both primarily affect domestic dogs (*Canis lupus familiaris*), other animals, particularly cats and wild carnivores, can also be infected [1]. *Dirofilaria immitis*, which has a worldwide distribution, parasitizes the pulmonary artery and the right chambers of the heart, leading to canine cardiopulmonary dirofilariosis, commonly known as heartworm disease. In contrast, *Dirofilaria repens* is found in subcutaneous tissues, causing subcutaneous dirofilariosis, and is endemic in Europe, Asia, and Africa, but not in the Americas [2]. Regardless of the adult parasites’ location, their offspring, the microfilariae, circulate in the bloodstream of competent hosts and can be transmitted to mosquito vectors, where they develop into the infective stage [2].

Both parasites have important zoonotic implications, as they may infect humans, especially in hyperenzootic areas, causing pulmonary, subcutaneous, or ocular dirofilariosis [3]. Although humans are accidental and in principle dead-end hosts for these parasites, these infections are considered emerging, as cases have increased in numbers in the last few decades [1].

The distribution of *Dirofilaria* species in Europe is expanding from the southern endemic areas to central and northern countries that were, until recently, considered free of infection [4]. In Greece, the prevalence of infection in dogs is higher in the northern regions and appears to be increasing in the south, where it was, until a few years ago, very low [5,6,7,8,9]. It has been documented that the prevalence of infection in dogs in a given area may predict the rate of infection of other permissive hosts, e.g., cats [6,10,11]. Furthermore, seroepidemiological studies of humans living in endemic areas have revealed seroprevalence rates similar to those recorded in dogs from the same regions [1].

As expected, most human cases in Europe have been recorded in Mediterranean regions; nonetheless, a few cases have originated in central and northern countries, e.g., Austria, Germany, Czechia, Slovakia, Poland, Belgium, and Finland [2,7]. Several human cases have been reported in Greece, involving pulmonary, ocular, and subcutaneous parasite localizations [12,13]. Other than the occasionally reported cases, no epidemiological study (e.g., serological surveys) of the population has been ever conducted to elucidate the epidemiology of human dirofilariosis in Greece. In this context, and within the collaborative and interdisciplinary-approach concept of One Health, the present study aimed to record, for the first time, the prevalence of dirofilariosis in dogs and humans living in Thrace, a hyperenzootic region in northeastern Greece [8,14].

## 2. Materials and Methods

### 2.1. Study Area

**Figure 1:**
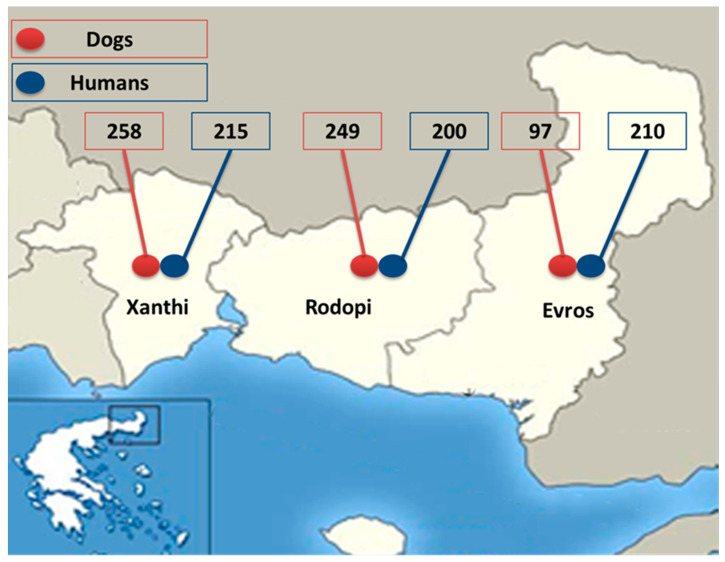
The geographic area of Thrace and the number of dog and human samples examined for *Dirofilaria* infection from the regional units, i.e., Xanthi, Rodopi, and Evros.

The geographical region of Thrace in northeastern Greece (Figure 1) was selected for this comparative study of *Dirofilaria* spp. infection in dogs and humans, based on previous data showing it had the highest prevalence of heartworm infection in dogs in the country [8,14]. Thrace (41°74′ N–40°73′ N, 26°63′ E–24°46′ E) is divided into three regional units, i.e., Xanthi, Rodopi, and Evros (Figure 1), and is characterized by extensive mountain ranges to the north, lowlands in the coastal zone, and the flow of two large rivers, Nestos and Evros. Approximately 31.3% of Thrace is covered by forests, while its main geological characteristic is wide wetland complexes of freshwater lakes and coastal lagoons, most of which are protected at national and European levels, as they are included in the Ramsar Convention on Wetlands [15,16]. In the lowlands and coastal areas, the climate is Mediterranean and temperate, with milder weather than in the mountains, and with high humidity due to frequent rainfall. The average temperature in the region is 14–16 °C [16].

### 2.2. Dog Samples

Dog blood samples were collected during routine clinical examinations. The animals included in this study fulfilled the following criteria: (i) they were living permanently in one of the regional units of Thrace, (ii) they did not receive regular preventative treatment with macrocyclic lactones against dirofilariosis, (iii) they had lived through at least one full mosquito activity period (MAP, May to October), and (iv) they were older than 12 months and at least 9 months had passed since the end of the MAP, if they had lived through only one. The written informed consent of the dog owner or the licensing of the municipal authorities, in the case of stray dogs living in shelters, was also a prerequisite.

Overall, 604 dogs were examined, specifically 258, 249, and 97, from the regional units of Xanthi, Rodopi, and Evros, respectively (Figure 1). A questionnaire was filled out for each dog, including information regarding the region of residency, lifestyle (outdoor/indoor/indoor–allowed outdoor), sex, age, and hair length. For each subject, 2 mL of blood was collected in an EDTA tube from a peripheral vein (cephalic or jugular) and kept refrigerated (4 °C) until examination.

### 2.3. Human Samples

A total of 625 human samples were collected from residents of the three regional units of Thrace, i.e., 215, 200, and 210 samples from Xanthi, Rodopi, and Evros, respectively (Figure 1). The human sera were obtained from blood samples collected with the individual’s written consent, from subjects living in one of the three regional units of Thrace. Humans of all ages and genders who were clinically healthy and who came to the General University Hospital of Alexandroupolis for a regular health check or as blood donors were included in the survey. After collection, the sera were stored in an Eppendorf tube at −20 °C until examination. Each study participant filled out a questionnaire, including information regarding their age, sex, and residency area. The collected human data were coded and stored anonymously, according to data protection legislation.

### 2.4. Sample Analysis

Each dog blood sample was examined by the modified Knott’s method for the detection of microfilariae [17]. The identification of microfilariae was performed under a light microscope at 100× and 400× magnification, on the basis of morphometric and morphological features [17,18]. The detection of the *D. immitis* antigen was performed by the commercial serological test DiroCHECK^®^ (Zoetis, Parsippany, NJ, USA).

The serological examination of the human blood samples was performed by Western blot analysis, separately for *D. immitis* and *D. repens* antigens, using parasites collected from canine cases of cardiopulmonary and subcutaneous dirofilariosis, respectively. For the development and standardization of the method, positive human sera from confirmed cases and negative control sera were used.

**Figure 2:**
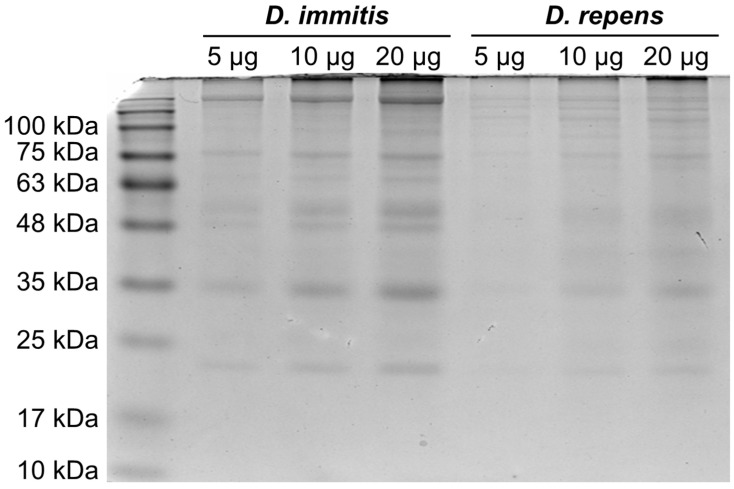
*Dirofilaria immitis* and *Dirofilaria repens* proteins’ quality and quantity estimation by analysis of 5 μg, 10 μg, and 20 μg in 10% Tris-glycine gel.

The parasites were homogenized per species in PBS supplemented with protease inhibitors (PMSF 100 μg/mL, Leupeptin 0.5 μg/mL, Aprotonin 0.5 μg/mL, and Pepstatin 1 μg/mL; Sigma Aldrich, St. Louis, MO, USA). Following homogenization, Triton X-100 at a 1% final concentration was added, and the samples were rapidly frozen at −80 °C, thawed, centrifuged at 10,000× *g* for five minutes, and the supernatant was collected and kept at −80 °C. The protein concentration of the lysates was calculated with a BCA assay kit (23225, Thermo Scientific, Waltham, MA, USA). The quality and quantity of the isolated proteins were estimated by loading 5 μg, 10 μg, and 20 μg from each species sample onto a 10% Tris-glycine gel (Figure 2). For the detection of *Dirofilaria*-specific antibodies, a quantity of 20 μg of protein from each parasite was selected and separated into 12% gels. The proteins were transferred onto polyvinylidene difluoride (PVDF) membranes (Immobilon, Darmstadt, Germany), and 5% non-fat dry milk was used to block non-specific binding. The sera were diluted 1:50 in a blocking buffer and incubated overnight at 4 °C. Following three washing steps, the membranes were incubated with an anti-human IgG HRP-conjugated secondary antibody (1:5000, 32935S, Cell Signaling, Danvers, MA, USA) and once again washed three times. Protein bands were detected using an ECL chemiluminescent substrate (34577, Thermo Scientific, Waltham, MA, USA) and a ChemiDoc MP Imaging System (BioRad, Hercules, CA, USA). The sera were considered positive for each parasite species when specific bands were detected, at 17–22 kDa for *D. immitis* and at 43–70 kDa for *D. repens*, respectively [19,20].

### 2.5. Statistical Analysis

The sample sizes for both the dog and human samples met the recommended threshold, indicating that to detect the difference in proportions with 95% confidence and 80% power, 91 samples were required [21]. The statistical analysis was performed using the chi-square test of independence between the infection status for *Dirofilaria* spp. (positive or negative result for any of the *Dirofilaria* species examined) and a variety of variables [22]. Specifically, for dogs, the variables examined were breed, gender, age, hair length, lifestyle, and area of residency; for humans, the variables examined were sex, age, and area of residency. The level of significance was set at *p* < 0.05.

## 3. Results

### 3.1. Dog Samples

Overall, 177 (29.3%) dogs were found to be infected with at least one species of *Dirofilaria*. Of the 604 dogs examined, 173 (28.6%) were positive for *D. immitis* by at least one of the examination methods (serology and Knott’s test). More specifically, 87 (50.3%) samples were positive for both methods, while 84 (48.6%) were positive for serology only, and 2 (1.2%) samples for Knott’s test only. Moreover, in seven (1.2%) samples, *D. repens* microfilariae were found in the Knott’s test. A mixed infection (*D. immitis* antigen or/and microfilariae and *D. repens* microfilariae) was found in six dogs. The results of the Knott’s tests and the serological examinations of the dog samples are presented in detail in Table 1.

At the regional unit level, the number of dogs found positive for *D. immitis* by serology and/or Knott’s test were 81 (31.4%), 57 (22.9%), and 35 (36.1%), in Xanthi, Rodopi, and Evros, respectively. Furthermore, *D. repens* microfilariae were detected in one (0.4%) dog in Xanthi and in six (6.2%) in Evros (Table 1).

### 3.2. Human Samples

**Figure 3:**
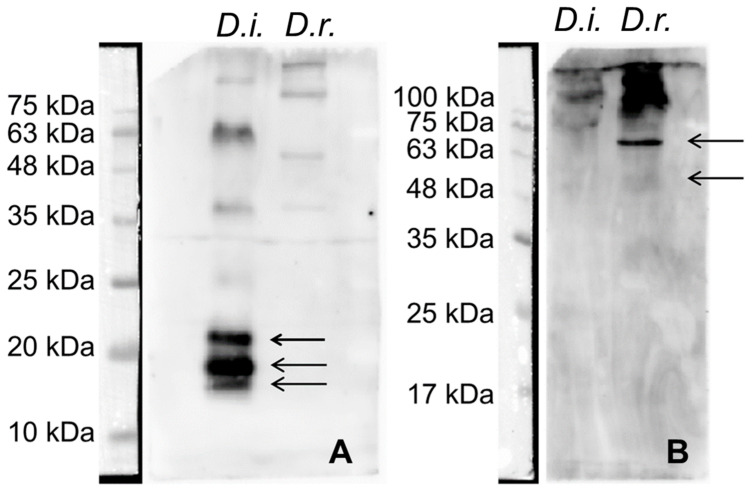
Western blot analysis of human sera showing bands between 17 and 22 KDa (indicative of *Dirofilaria immitis*, panel (**A**)) and between 43 and 70 kDa (indicative of *Dirofilaria repens*, panel (**B**)). The respective bands are indicated by arrows.

Based on the results from the method standardization using positive and negative control samples, and in accordance with the literature, human sera were considered positive for *D. immitis* when bands between 17 and 22 kDa were detected, and positive for *D. repens* when bands between 43 and 70 kDa were observed (Figure 3) [19,20,23].

Overall, 42 (6.7%) samples were positive on the Western blot analysis for *Dirofilaria* spp.-specific IgG antibodies, i.e., 24 (3.8%) for *D. immitis* and 18 (2.9%) for *D. repens*. In more detail, 5 (2.3%) samples were positive for *D. immitis* and 10 (4.7%) samples were positive for *D. repens* in Xanthi, 9 (4.5%) and 6 (3%) in Rodopi, and 10 (4.7%) and 2 (1%) in Evros, respectively (Table 2).

### 3.3. Statistical Analysis

The statistical analysis of the dogs’ results included 546 of the total 604 animals, due to insufficient data for 58 of them. Factors, such as gender [Xχ^2^ (1, N = 546) = 5.21, *p* = 0.0022], lifestyle [χ^2^ (2, N = 546) = 18.23, *p* < 0.001], and area of residency [χ^2^ (2, N = 546) = 10.57, *p* = 0.005], were associated with *Dirofilaria* spp. infection, while hair length and age were not (*p*-value > 0.05) (Table 3). Male dogs were more likely (OR = 1.55) to be infected than female dogs, and dogs living in the prefecture of Evros were more likely to be infected than dogs living in Rodopi (OR = 2.03) or Xanthi (OR = 1.15). Finally, dogs living outdoors had an increased risk of infection compared to dogs living indoors (OR = 2.4) or to indoors–allowed outdoors (OR = 2.27) (Table 3).

The statistical analysis of the human samples revealed no significant statistical correlation between demographic factors (sex, age, and area of residency) and the risk of infection (*p*-value ≥ 0.05).

## 4. Discussion

This study is the first to comparatively investigate the prevalence of *Dirofilaria* spp. infection in both dogs and humans in a hyperenzootic area of Greece. Additionally, it represents the first serological screening for *Dirofilaria* spp. infection in the human population of Greece.

The hyperenzootic profile of the selected study area was confirmed by the present results, with an overall infection rate in dogs of 28.6% and the highest prevalence percentage of 36.1% recorded for the regional unit of Evros. On this basis, the results of the present study provide evidence regarding the rate of human contact with parasites of the genus *Dirofilaria* in an area of high infection pressure.

The hyperenzootic character of northern Greece for *D. immitis* has repeatedly been demonstrated by surveys of dog populations. The percentage of infection in dogs varies from 6.1% up to an extreme of 68%, which was found in dogs from the easternmost town of Didimoticho in Evros [5,14,24,25,26,27]. This epizootiological status is associated with the geomorphology of northern Greece and the agricultural profile of the area. Indeed, northern Greece holds most of the wetlands of the country, 91% of the total rice fields, and 68.5% of the farm cattle population at the national level [5,28,29]. These conditions may favor mosquito development and their populations’ abundance and stability [30,31]. This hypothesis is confirmed by entomological surveys that have demonstrated that the *Aedes* spp. population in eastern Macedonia and Thrace is 10 times larger than the corresponding population in southern or in western Greece [32]. Furthermore, the mosquito species *Culex pipiens* and *Aedes caspius*, proven vectors of canine dirofilariosis, are the dominant mosquito species in northern Greece [32,33,34,35].

None of the dogs examined herein presented with clinical evidence of heartworm disease or subcutaneous dirofilariosis at the routine clinical examination. However, heartworm disease is one of the most severe parasitic diseases in dogs and can be fatal. The pathogenesis of *D. immitis* is mainly related to pulmonary hypertension that leads to congestive heart failure [2]. Common clinical signs include chronic cough, respiratory distress, weakness, ascites, abnormal cardiac sounds, and sudden death [2,36,37]. *Dirofilaria repens* infection is de facto less severe. When clinical sings occur, they usually manifest as skin nodules that may develop in various sites, ranging from 0.5 to 3 cm in diameter [36].

Among the factors examined herein, the sex, lifestyle, and area of residency were found to be associated with a higher risk of infection in dogs. Although it has been observed that in hyperenzootic areas the sex and activity level of dogs do not play a role in the risk of infection [2], male dogs are used more often as guards than female dogs, increasing the time spent outdoors under the risk of mosquito bites. Indeed, dogs living exclusively outdoors were found to have an increased risk of infection compared to dogs living indoors or indoors with outdoor access. Dogs living in the regional unit of Evros had the highest probability of infection compared to the other two regional units of this study. This is in accordance with previous data indicating Evros as the area with the highest prevalence of dog infection in Greece [8,14]. Although age was not found to be a statistically significant factor for the risk of infection, dogs over 7 years of age showed a higher percentage of infection. This observation is common in canine dirofilariosis, which is a chronic condition, thus occurring more prevalently in older dogs as they accumulate exposure to infection over the years.

Human dirofilariosis is considered a sporadic zoonotic disease. However, in the last decades, the number of reported human cases have increased [1]. This may be attributed to a factual increase in incidences of human infection, due to various factors that promote the spreading of vector-borne diseases (VBDs), but also to the enhancement of awareness in the medical community. The factors that are linked to the expansion and increase in VBDs [38], primarily among natural hosts and subsequently among accidental hosts, in this case, dogs and humans, respectively, are i. climate change, especially global warming and extreme meteorological phenomena (e.g., floods), ii. land use changes, which influence vectors and reservoir hosts’ biology and behaviors, and iii. the intensified movement of humans, animals, and goods that promote vector and host spreading [39,40].

In Europe, most human cases are reported in Italy, followed by France, and then Greece [1]. However, cases in central and northern European counties have also received publicity in the recent years [3,4]. *Dirofilaria repens* is the primary agent of human dirofilariosis in Europe and has been found in various sites of the human body, with the predominant region being the head, and particularly the subcutaneous tissues and the eye (eyelid, periorbital region, and subconjunctiva) [1,3]. Although humans are not the preferred host for *D. repens*, there have been several cases where the parasites fully matured and produced microfilariae within a human host. To date, there are at least 24 cases of *D. repens* microfilaremia in humans [41,42,43].

On the other hand, *D. immitis* infections in humans are less common in Europe [1,3]. *Dirofilaria immitis* typically migrates to the pulmonary arteries, where it is usually destroyed by the host’s immune system, generating a granulomatous nodular lesion in the lung parenchyma, known as “coin lesion”. This condition is usually asymptomatic and often discovered incidentally during imaging examinations. However, in some cases, the presence of the parasite can cause thoracic pain, cough, hemoptysis, low fever, and malaise [44]. Although the condition is generally self-limiting, the surgical removal of the lesion is the standard treatment, as malignancy is considered in the differential diagnosis [3]. *Dirofilaria immitis* has never been reported to have fully developed in humans, and there is no case of recorded microfilaremia.

The higher frequency of human *D. repens* infections compared to *D. immitis* infections may reflect the parasite’s better adaptation to human host, as evidenced by the occasional reproductive maturity that the parasites reach in humans, resulting in microfilaremia. In addition, the subcutaneous and ocular localization commonly associated with *D. repens* infections facilitates its diagnosis compared to the parenchymal and most often pulmonary localization of *D. immitis* [3].

Despite the notion that in humans *D. repens* is related to subcutaneous nodules and ocular localizations while *D. immitis* causes pulmonary nodules, both parasites have been found in many different localizations within the human body; thus, the localization of the parasite cannot by any means imply its species [1]. Furthermore, although the morphology of the cuticle of *Dirofilaria* may indicate the species, as *D. repens* displays a longitudinally striated cuticle while *D. immitis* does not, the parasites, when they are extracted from the organ of parasitism, are often already dead and destroyed to an extent, and thus morphologically altered. In many cases, the only reliable identification method is via molecular techniques (PCR) [3,13]. In fact, as reviewed by Pampiglione et al. [45], the morphological misidentification of parasites at the species level is rather common in the literature.

Human dirofilariosis, due to both *D. immitis* and *D. repens*, has been documented in Greece on several occasions [12,13]. However, in only a few instances of ocular and one case of subcutaneous dirofilariosis were the parasites unequivocally characterized by molecular means as *D. repens* [46] and *D. immitis* [47], respectively.

In contrast to the relatively straightforward diagnosis of *Dirofilaria* infections in dogs, diagnosis in humans is more complex. Indeed, in dogs, *D. immitis* infection is diagnosed by the combination of Knott’s method and a serological test. This is the recommended laboratory diagnostic procedure, providing very high diagnostic accuracy, as it covers cases of occult infection (infection in the absence of circulating microfilariae) and false-negative serological results (low levels of circulating antigens) [36,37]. Notably, both scenarios were encountered in the present study, as an occult infection was detected in 84 out of the 173 infected animals, and two animals had circulating *D. immitis* microfilariae with a negative serological test. For *D. repens*, there is no serological test, so Knott’s method is the only laboratory diagnostic approach, revealing an infection in cases with microfilariae circulation but missing occult infections [36]. On the other hand, diagnosis in humans, other than the surgical removal and identification of a parasite when detected, is not easy, as microfilaremia is very rare and has only been observed in some cases of *D. repens* infection. Similarly, due to the rarity of infections, there is no commercial serological test available for humans. Therefore, in-house ELISA and Western blot assays have been developed and applied in seroepidemiological surveys. In fact, serology is the only method by which to identify individuals who have recently been exposed to the parasites.

ELISAs developed for the detection of specific anti-*Dirofilaria* spp. antibodies have employed the somatic and excretory/secretory proteins of the parasites, as well as the proteins of their bacterial endosymbiont *Wolbachia*, as antigens [48,49,50,51]. The crude somatic or excretory/secretory antigens used in ELISA have the drawback of cross-reactions with other parasites that may infect humans, especially *Toxocara canis*, a canine nematode causing “visceral larva migrans”, one of the most common zoonotic infections in the world [1,52]. Western blot analysis, on the other hand, provides a reliable serological tool, with specific molecular weight bands indicating seropositivity for *D. immitis* and *D. repens* separately [3].

The seroprevalence in humans in Europe has been investigated for a few cases. In western Spain and in the Canary Islands, 9.3% and 6.4% of the population examined by ELISA (somatic antigen) were *D. immitis* positive, respectively [49,53]. In Portugal, a serosurvey by ELISA using a *D. immitis* somatic antigen and a *Wolbachia* surface protein as antigens showed a prevalence of 6.1% [54]. In Romania, Moldova, and Serbia, an ELISA with a somatic antigen was used, coupled with the detection of an anti-*Wolbachia* surface protein and Western blot. Taking into account the mixed infections, 7.4%, 14.8%, and 2.6% were positive for *D. immitis,* and 0.5%, 1.5%, and 2.3% showed *D. repens* seropositivity in the three countries, respectively [19,20]. These percentages are close to the prevalence found in the present study, i.e., 3.8% for *D. immitis* and 2.9% for *D. repens*. Interestingly, the percentage of *D. repens*-seropositive humans was higher than that of *D. repens*-microfilaremic dogs (1.2%), suggesting that the actual infection rate in dogs is much higher than what is revealed by the presence of circulating microfilariae. The age, sex, and area of residency of the individuals included in the present study were not associated with a higher risk of human dirofilariosis. Age in humans, as in dogs, is associated with a higher infection rate, due to repeated exposure to the parasite in an enzootic area [55]. However, the short lifespan of the parasites in a non-natural host clears the infection much sooner than in dogs, preventing chronic infections and, thus, restricting the time during which antibodies can be detected.

## 5. Conclusions

*Dirofilaria* spp. infection in dogs living in the geographic region of Thrace remains highly prevalent. The detected proportion of infection in humans was 23.4% of the respective canine infection in the same area. Knowing the rate of human contact with parasites in areas of high infection pressure is essential for implementing preventive measures, raising awareness among the medical community, and providing available data to promote and support the prompt and accurate diagnosis of human infections. By incorporating the “One Health” concept, the present study emphasizes the interconnectedness of human, animal, and environmental health.

Timely diagnosis and, more importantly, effective prevention in dogs are crucial for protecting the health of both animals and humans. This is especially vital given the anticipated rise in cases and the spread of parasites due to climate change, increased animal travel, and the presence of invasive mosquito species. The monitoring and control of *Dirofilaria* infections have acquired additional significance recently, due to the development of resistant strains of *D. immitis* to macrocyclic lactones, i.e., the only drug molecules used for heartworm prevention [56,57]. Furthermore, medical doctors should be adequately informed about *Dirofilaria* infections in humans for its accurate and prompt diagnosis that, in some cases, may prevent unnecessary medical procedures (e.g., surgeries) for patients. In this context, sensitive and easy-to-perform routine-level serological methods should be developed for diagnosis in humans. Furthermore, these infections should become notifiable diseases in all enzootic/endemic countries, as is currently the practice in some eastern European countries, such as Ukraine, Belarus, and Russia, where a high number of cases have been reported in recent years [58]. This measure, in combination with regular epizootiological/epidemiological screenings of the dog and human populations for *Dirofilaria* spp., is imperative for the surveillance of these important zoonotic infections.

## Figures and Tables

**Table 1:** Dogs found positive by different examination methods for *Dirofilaria immitis* and *Dirofilaria repens* in the geographical region of Thrace, and in each separate regional unit within Thrace.

	Thrace (n = 604)		Xanthi (n = 258)		Rodopi (n = 249)		Evros (n = 97)	
Examination Method	D.i.	D.r.	D.i.	D.r.	D.i.	D.r.	D.i.	D.r.
Knott	86 (14.2%) *	7 (1.2%) *	45 (17.4%)	1 (0.4%)	20 (8%)	0	21 (21.6%) *	6 (6.2%) *
Serology	171 (28.3%)	-	80 (31%)	-	56 (22.5%)	-	35 (36.1%)	-
Knott or/and Serology	173 (28.6%) *	7 (1.3%) *	81 (31.4%)	1 (0.4%)	57 (22.9%)	0	35 (36.1%) *	6 (6.2%) *

D.i. = *Dirofilaria immitis*; D.r.= *Dirofilaria repens*; * mixed infection in 3 animals.

**Table 2:** Seropositive (IgG) human samples by Western blot analysis for *Dirofilaria* spp.

Regional Unit	Seropositive Samples Per Parasite		
	*Dirofilaria* spp.	*D. immitis*	*D. repens*
Thrace (n = 625)	42 (6.7%)	24 (3.8%)	18 (2.9%)
Xanthi (n = 215)	15 (7%)	5 (2.3%)	10 (4.7%)
Rodopi (n = 200)	15 (7.5%)	9 (4.5%)	6 (3%)
Evros (n = 210)	12 (5.7%)	10 (4.7%)	2 (1%)

**Table 3:** Statistical analysis of the variables recorded for the dogs examined for *Dirofilaria* spp. in the hyperenzootic area of Thrace, Greece.

Variable	*Dirofilaria* Positive	*Dirofilaria* Negative	χ^2^ Test/Fisher Test (*p*-Value) Odds Ratio
Sex			
Male (199)	70 (35.2%)	129 (64.8%)	5.21 (**0.022**)
Female (347)	90 (25.9%)	257 (74.1%)	Odds ratio = 1.55
Age *			
≤3 (255)	67 (26.3%)	188 (73.7%)	4.92 (0.089)
3−7 (215)	63 (29.3%)	152 (70.7%)	
>7 (76)	30 (39.5%)	46 (60.5%)	
R.U. **			
Evros (97)	36 (37.1%)	61 (62.9%)	10.57 (**0.005**)
Rodopi (249)	56 (22.5%)	193 (77.5%)	Odds ratio = 2.03
Xanthi (200)	68 (34.0%)	132 (66.0%)	Odds ratio = 1.15
Lifestyle			
Outside (272)	102 (37.5%)	170 (62.5%)	18.23 (**<0.001**)
Inside (10)	2 (20.0%)	8 (80.0%)	Odds ratio = 2.40
In and out (264)	56 (21.2%)	208 (78.8%)	Odds ratio = 2.27
Hair length			
Short (243)	79 (32.5%)	164 (67.5%)	3.00 (0.223)
Medium (225)	57 (25.3%)	168 (74.7%)	
Long (78)	24 (30.8%)	54 (69.2%)	

* Age group (age in years); ** R.U.: regional unit; *p*-value in bold: statistically significant.

## Data Availability

The raw data supporting the conclusions of this article will be made available by the authors upon request.
